# Gleanings from Our Exchanges

**Published:** 1872-05-01

**Authors:** 


### Gleanings from Our Exchanges.

Tincture of Iodine in Vomiting.—Schneider, of Offenbach, in a patient who was taken with salivation and vomiting after intermittent fever, which vomiting continued for several weeks incessantly, in spite of various remedies, administered the tincture of iodine in doses of ten drops on sugar thrice daily, and obtained after the first dose the cure of these symptoms. In the same way, too, he treated with great success a soldier laboring under intermittent fever, which had resisted all the antipyretics from quinine to arsenic. The intraparenchymatous injection of iodine in the treatment of hypertrophied tonsils has been used with great success by Dr. Rumbold. The liquid injection is composed of ten centigrammes of iodine, 2·40 grammes of iodide of potassium, and thirty grammes of water. After each injection, slight inflammation succeeds, which soon disappears. Twelve to seventeen injections are required to restore the tonsils to their normal size, two injections weekly. This practice has the advantage of retaining intact the substance of the tonsils.—*Doctor.*

Treatment of Venereal Ulcers by Dr. Hemard.—In the *Med.-Chirurg. Centralbl.*, 1871, Dr. Hemard asserts that for the last twenty years he has obtained the cure of soft and hard sores by simply irrigating the parts with cold water. A vessel of cold water is fixed to the walls of the room, at such a height that the water which comes from it through a tube, attains a certain force of projection. The patient has no more to do but to wash his ulcers every three or four hours under this stream; in a few days the ulcer becomes clean, and quickly heals. All other treatment is superfluous. In ulcers of the prepuce, which are out of sight, after irrigation, a little starch flour is introduced. When the superficies of the ulcer has lost its characteristic aspect, a stratum of collodium is painted on it, and it soon heals.—*Ibid.*

Croup.—M. M. Mouttet and Rosiere, of Montpellier, report that they have cured croup, when tracheotomy was refused by the parents as a useless cruelty, by repeated insufflations of powdered nitrate of silver. The application was at once followed by the detachment and expulsion of false membrane, and was repeated a quarter of an hour after with a similar result. Three or four hours later another insufflation was followed by so much relief that hope was entertained, and the child from this time made a steady recovery.—*Ibid.*

A Case of Quadruplets.—Mr. Lowndes, *British Medical Journal*, reports a case of quadruplets. The first child was a female and living, the remaining three were either putrid or in a state of incipient decomposition.

Varioloid after Vaccination.—Dr. Richard A. Clemann reports (*Phila. Med. Times*) a case of mild varioloid occurring in an Irish boy 17 years of age, within three months after successful vaccination. The boy was living in a small room in which a man was sick with a severe case of confluent small-pox, and passed much of his time with him.

Nitrate of Silver in Bed-Sores.—Betz regards nitrate of silver as the best remedy for bed-sores. Instead of making use, however, of lint dipped in the solution of lunar caustic, he prescribes an ointment composed of five decigrammes of the nitrate of silver, fifteen grammes of lard, and thirty of wax; which he spreads on linen, and applies to the sores, taking care that the piece is rather larger than the sore. This is repeated morning and evening—*Ibid.*

Hæmoptysis in Phthisis.—In one of the papers on "Phthisis Pulmonalis," which recently appeared in the columns of the *Medical Press and Circular*, Dr. R Locke Johnson speaks of the *increased tendency* of patients to hæmoptysis, during the spring time and the early summer time. Having for some years past made observations on this point, he has occasionally been enabled to retard altogether or to lessen this lesion, by attending closely to the secretions, the skin, and the pulse of his patients.—*Ibid.*

Pitting from Small-Pox.—A writer in the *Brit. Med. Jour.* states that he considers *pressure* the main point to be relied on—by lessening the vascularity of the part and preventing the maturation of the pustules—in cases where pitting from small-pox is feared. Strapping in parts where it may be possible to do so answers this purpose fully, and the next best thing to strapping is to employ xylonite, a collodion which, although somewhat elastic, quickly forms—when employed over the cuticle—a dense and tight skin, more dense, tight, and tenacious than the collodion of the *Pharmacopœia*. It acts like strapping, and produces abortion of the pustules. To parts of the face where strapping cannot be employed with advantage, xylonite will be found to be the next best mode of treatment. The late Dr. Graves remarked that in a patient whose knee had been strapped for a surgical operation being attacked by small-pox, the portion over which the strapping extended was free from even the rash.—*Ibid.*

Turpentine is said to give relief in phosphorous poisoning.

Alcoholic Paraplegia.—Dr. Wilks, of Guy's, in a paper in the *Lancet*, endorses the conclusions lately published by Dr. Handfield Jones, in reference to the production of epilepsy and other nervous diseases by the abuse of alcohol. Dr. Wilks has met with cases where the alcohol, acting chiefly on the spinal cord, made paralysis the leading symptom, and he combats in his own trenchant style the notion that it is not safe to suddenly leave off the accustomed stimulus. "No harm," he says, "but only good will ensue from its withdrawal." He considers that the same rule should apply to all persons.—*Ibid.*

Early Diagnosis of Typhoid.—Dr. P. W. Latham, in the *Lancet*, insists on the value of the thermometer in diagnosing between those slight feverish attacks which are so common. If a person previously quite well begins to feel uneasy, and in the evening we find his temperature about 109.4°, or 101° F., falling the next morning and rising again in the evening and approximately following the above course the disease may be diagnosed as typhoid with tolerable certainty.

On the other hand, the disease is not typhoid fever if (1) on the second, third, or fourth evenings the temperature approximates even to normal (98.6° F.); (2), if during the first two days the temperature rises to 104° F.; (3), if between the fourth and sixth days the evening temperature of a person under middle age does not reach 103°; (4), if the temperature on two of the first three evenings is the same; or (5), if it is the same on the second or third mornings.

The Use of Glycerine as a Solvent in Hypodermic Injections.—Dr. M. Rosenthal calls attention, in the *Wiener Medizinische Presse* for January 7, 1872, to the power which glycerine possesses to dissolve various of the substances which are ordinarily used in hypodermic medication. Its solvent powers are greater than those of water, and are very much increased by heat. Thus, a fluid drachm of glycerine, when heated, will readily dissolve twenty grains of the sulphate of quinia, from ten to twelve grains of the acetate or muriate of morphia, and ten grains of the extract of opium. Morphia may be added to a solution of quinia in glycerine without causing a precipitate. It will also dissolve from half a drachm to one drachm of the iodide or bromide of potassium, and four grains of corrosive sublimate. These substances are not precipitated as the liquid cools; on the contrary, the solution will remain clear and fit for use during at least a year.—*Ibid.*

Cod-Liver Oil in Whooping-Cough.—J. Prestwich, L. C. P. etc., has tried cod-liver oil in whooping-cough with decided benefit.—*Ibid.*

Subcutaneous Injection of Morphia in Cholera.—Dr. A. Werry states that he has tried the subcutaneous injection of morphia in cholera with the happiest results.—*Ibid.*

A Visible Striation of the Normal Crystaline Lense.—Mr. John Tweedy describes certain "dark radiating lines" which he observed in the crystaline lense, by oblique illumination. He has seen them in every case which he has examined since he first observed them. To see them, he uses a bi-convex lense, of about 2¼ in focus, and throws the light of an Argand gas burner obliquely on the eye of the patient, concentrating the light on the lense. He then looks very obliquely, "at an angle of about 130° or 140°," on the plane of the anterior surface of the lense. It is quite difficult to get a view of the lines at first, but when they are seen they are quite distinct.—*Ibid.*

The Application of the Diaphason to Auscultation.—W. H. Griffiths, Ph. D., L. R. c. P. & S. Ed., suggests the use of the ordinary tuning fork in place of percussion, claiming that the results are far superior. He attaches the tuning fork to an end something like a stethoscope; from the "stem" which connects the prongs of the fork to the dome-like end, a handle passes out horizontally. When used, the handle is held in the hand, the dome-like end is applied to the part of the body which is to be investigated, and the instrument sounded as in its ordinary use. He claims that results far superior to percussion can be obtained by this instrument, and recommends it to other physicians for trial.—*Ibid.*

Treatment of Pulmonary Emphysema by Artificial Expiration.—Dr. J. B. Berkart, recognizing that the difficulty in pulmonary emphysema arises from an inability to expel the air from the lungs, describes a method of treating the disease by withdrawing air from the lungs by artificial means, which he claims to have found very successful in his hands. The apparatus which he uses consists of a mask made to fit closely over the mouth and nostrils, so as to exclude the air. On the front of this mask is a chamber from which a tube leads to an air pump; there are valves also in the chamber, one which can be closed by the finger to admit air during inspiration, and another to allow the air to pass out during expiration. The instrument is used in the following manner: The mask is fitted firmly to the face; on inspiration the air enters of itself, at the end of expiration the inspiratory valve is closed by the finger and the air-pumps worked a moment; thus withdrawing the air from the lungs more completely. The finger is then withdrawn and the lungs allowed to fill again. This process is continued for ten or fifteen minutes, twice a day. The patients experience almost instant relief.

Bronchorrhœa and great dyspnœa are no contra indications; only in the latter condition the operation may have to be interrupted.—*Lancet.*

No. 1. Bowel Lesion of Typhoid Fever.—Dr. T. J. Maclagan contributes an article on this subject, of which we present an abstract. In regard to the physiology of the agminated and solitary glands, he argues that they are true absorbent glands which are somehow engaged in the elaboration of the fluid which is carried away by the lacteals. He founds his belief on the following facts: first, the appearance of the glands. They look like lymphatic glands under the microscope, and they have been injected by Bruck through the lacteals. Their position also supports this view, if their object was to supply anything to assist in the digestion of the food they would be higher up in the intestine. The sphincter illi probably has for its function the narrowing or temporary occlusion of the ilium, and consequent detention of the chyle in contact with these glands.

The period of life at which they are most active is adolescence and early manhood. They diminish from middle life to old age; hence, the doctor argues, that they supply something which the reproductive organs appropriate to themselves.

He opposes the opinion that the bowel lesion is due to the effort of the glands to eliminate the fever poison from the system; stating that the whole facts in favor of this view are, (1), that inflammation of these glands is an invariable accompaniment of typhoid fever, and (2), that the discharges from the bowels largely contain the poison of that disease.

To this he opposes the following reasons: 1. "The glands are possessed of absorbent functions only, and are in no sense eliminatory.

2. "Even if they did possess eliminatory functions it is very improbable that the work of elimination would be thrown to so large an extent on the glands at the lower extremity of the ilium, while those high up escaped altogether.

3. "Were they eliminatory, they would not probably be destroyed (as they are in typhoid fever) while performing a duty so necessary to the well being of the body.

4. "Were the elimination of the poison by these glands nature's way of relieving the system, the deposition by them of the morbid material ought to be followed by an amelioration, of the general symptoms of the disease. We know that the contrary holds good, and that the bowel lesion increases rather than diminishes the general febrile disturbance.

5. The opinion that the poison is thus eliminated is at variance with the fact that mild cases of typhoid fever do occur in which the glandular lesion terminates in resolution, and in which the so-called morbid deposit is absorbed into the system."

It will be seen that, while holding very different views from Beale in regard to the physiology of the glands, the doctor arrives at almost the same conclusions with him as to the part they play in elimination.

He goes on to state that the sloughs and discharges from these glands carry the contagion of the disease, and that they may carry the disease from glands which are affected "*to the unaffected glands* with which they *come in contact in their progress down the intestines*," in fact that there are two forms of bowel lesion, a *primary* and a *secondary*.

His reasons for this belief are:

2. That it is consistent with analogy that the discharges from a specifically inflamed part applied to a like organ in a state of health should reproduce the specific inflammation in this organ.

The individual lesions are of two kinds; in one, the *plaques dures*, the process commences in the interior of the gland and the mass sloughs out bodily; in the other, the *plaques molles*, the process of destruction commences by a superficial ulceration which eats its way through the mucous and sub-mucous coats and may even penetrate the peritoneal. The only satisfactory explanation of this difference is that the *plaques molles* is a secondary inflammation.

3. The glands of the lower part of the ilium suffer more than those higher up.

4. The extent of the intestinal lesion as revealed by examination after death bears no relation to the severity of the abdominal symptoms during life. When there is a severe diarrhœa, the discharges are carried off and do a minimum amount of harm; when the bowels are confined, the discharges remain in contact with the glands longer and cause more injury. Hence perforation is more liable to occur in mild cases.

Such are the main points of the paper. We have space only to glance at his treatment, which consists in giving rest as far as possible to the affected glands by supplying nourishment that will be absorbed by the upper part of the intestinal canal; milk is best, it should not be given *ad libitum* but in definite quantities and at stated times.

The bowels are to be kept in a natural condition; looseness should not be checked unless the stools are so frequent or copious as to affect injuriously the patient's strength; as a general rule, if the stools do not exceed three a day they should not be checked. Constipation should be obviated by mild laxatives, he prefers phosphate of soda.—*Ibid.*

The Bruit du Diable.—M. Duchek (*Lancet*, February 8, 1872) thus explains this murmur: The jugular vein behind the insertion of the sterno-cleido-mastoideus is wider than elsewhere, forming the bulbus. Beneath this bulbus are valves in the narrowest part of the vein. These valves have a most important influence upon the whole circulation of the blood. The pressure in the thorax being too high, they approach one another, and in this way oppose the further entrance of blood into the thorax. The part above them must be consequently dilated, as we remark it in cases of stagnation in the heart. The jugular vein being distended, and jugulum growing gradually more shallow, the impulse of the blood makes these valvulæ vibrate, and causes the murmur. When the blood flows slowly the impulse is too weak, and no murmur is to be heard. The murmur arises when the valvulæ are half opened and put into vibration by a sufficient rush of blood. If the pressure in the thorax increases, by valvular failures, emphysema of the lungs, etc., the stream flows slowly; and therefore we do not find these murmurs attending disorders of the intra-thoracic organs; and hence the general view that this bruit excludes insufficiency of the valvula mitralis. Another consequence of this fact is that these murmurs are not to be heard in persons affected by anæmia when they become the subjects of pneumonia or exudative pleurisy, and that they reappear at recovery. The two necessary requisites, then, are a speedy circulation of blood and a normal pressure of blood in the thorax. This view affords us an explanation of the murmur growing stronger when the respiration is more hurried.—*Phila. Med. Times.*

Carbolic Acid, Professor Gross says in a clinical lecture, was a fanciful preparation that had its day.

Dr. Caspari, of Horn, has for the last 25 years successfully prescribed the tincture of iodine in cases of obstinate vomiting.

